# Diabetes and the risk of bladder cancer subtypes in men and women: results from the Netherlands Cohort Study

**DOI:** 10.1007/s10654-024-01100-0

**Published:** 2024-03-16

**Authors:** Piet A. van den Brandt

**Affiliations:** 1 GROW- School for Oncology and Reproduction, Department of Epidemiology, Maastricht University Medical Centre, PO Box 616, 6200 MD Maastricht, The Netherlands; 2 CAPHRI- School for Public Health and Primary Care, Department of Epidemiology, Maastricht University Medical Centre, Maastricht, The Netherlands

**Keywords:** Bladder cancer, Diabetes, Cohort study, Prevention

## Abstract

Meta-analyses have shown modest positive associations between diabetes mellitus (DM) and bladder cancer risk, but results are heterogeneous. This might be due to lack of distinction between bladder cancer subtypes, between sexes, and possibly between Type 2 and Type 1 DM (T2DM and T1DM). The relationship of T2DM (and secondarily T1DM) characteristics with risk of bladder cancer subtypes (invasive versus noninvasive) was investigated in the Netherlands Cohort Study. In 1986, 120,852 men and women aged 55–69 years provided information on DM and lifestyle data. After 20.3 years of follow-up, multivariable case-cohort analyses were based on 1020 invasive and 1088 noninvasive bladder cancer cases, and 4267 subcohort members with complete data on DM and confounders. While T2DM was not associated with noninvasive bladder cancer, it was statistically significantly associated with invasive bladder cancer risk: the multivariable-adjusted was HR = 1.57 (95% CI 1.04–2.37), comparing participants with T2DM versus without DM. The association was only significant in women, and women showed a stronger association [HR = 2.19 (95% CI 1.10–4.34)] between T2DM and invasive bladder cancer than men [HR = 1.42 (95% CI 0.88–2.30)]; interaction by sex was nonsignificant. Associations were stronger positive in those whose age at diagnosis of T2DM was 55+ years, and in those diagnosed with T2DM less than five years before baseline. T2DM participants using antidiabetic medication had higher invasive bladder cancer risk than those without DM. Exploratory age-sex-adjusted analyses suggested a positive association between T1DM and invasive bladder cancer, but this was based on few cases. These findings suggest that T2DM and possibly T1DM are positively associated with invasive bladder cancer risk.

**Supplementary Information:**

The online version contains supplementary material available at 10.1007/s10654-024-01100-0.

## Introduction

With approximately 573,000 new cases, bladder cancer was the tenth most commonly diagnosed cancer worldwide in 2020. Bladder cancer is approximately three times more often diagnosed in men than in women, and it is the sixth most common cancer in men worldwide. The highest incidence rates occur in Western and Southern Europe, and in North America [1]. Bladder cancer ranges from noninvasive tumors that recur to aggressive invasive tumors with much poorer survival rates. Due to its high rate of recurrence, bladder cancer has one of the most expensive lifetime treatments of all cancers, resulting in a burden of the health care system [2]. Primary prevention is therefore of great importance to reduce the burden of this disease. Apart from increasing age, tobacco smoking and certain occupational exposures (polycyclic aromatic hydrocarbons and aromatic amines) are established risk factors for bladder cancer [3].

In meta-analyses of cohort studies, a history of Type 2 diabetes mellitus has been associated with increased cancer incidence, most notably with pancreatic, liver and endometrial cancer [4]. Although many studies have been performed on diabetes and bladder cancer risk, and meta-analyses of case–control and cohort studies combined have shown a modest positive association [5, 6], recent results are inconsistent according to a recent systematic review [3]. However, in the latter review [3], no distinction was made between bladder cancer subtypes in the association with diabetes. In another meta-analysis, bladder cancer risk was inversely related with duration of diabetes mellitus, with the increased risk found in those diagnosed with diabetes less than 5 years ago [6].

The inconsistencies in the findings may be due to lack of distinction between subtypes of bladder cancer (e.g., invasive versus noninvasive subtypes), and possible differences between men and women in the associations of diabetes mellitus with bladder cancer. Furthermore, a distinction between Type 1 and Type 2 diabetes mellitus has not always been made in studies.

The association between a history of diabetes and the risk of bladder cancer (subtypes) was investigated among men and women separately in the Netherlands Cohort Study on diet and cancer (NLCS). The primary aim of the current investigation was to examine the association between Type 2 diabetes mellitus (T2DM) and risk of bladder cancer subtypes (invasive versus noninvasive), and evaluating various exposure aspects like T2DM duration, age at diagnosis of T2DM, and use of antidiabetic medication. Recently, an Italian cohort study found a positive association between Type 1 diabetes (T1DM) and bladder cancer risk (as well as T2DM) [7]. Thus, while the focus in the NLCS was on Type 2 diabetes mellitus (T2DM), a secondary hypothesis was that T1DM may also be related to bladder cancer risk; explorative analyses on T1DM were therefore also performed.

## Methods

### Study design and cancer follow-up

The NLCS started in September 1986 and included 58,279 men and 62,573 women aged 55–69 years [8]. At baseline, participants completed a mailed, self-administered questionnaire on cancer risk factors. The NLCS study was approved by institutional review boards from Maastricht University and the Netherlands Organization for Applied Scientific Research. All cohort members consented to participation by completing the questionnaire. For efficiency, we applied the nested case-cohort method [9], requiring only data-entry of questionnaires (which could not be scanned) of cases and a random subcohort. Following this method [8], cases were enumerated from the entire NLCS-cohort of 120,852 (numerator information of incidence rates), whereas the accumulated person-years at risk in the cohort were estimated using a subcohort (n = 5000; 2411 men and 2589 women), randomly sampled from the cohort immediately after baseline (denominator information). The case-cohort method implies that the persontime at risk is estimated through a sample of the total cohort, instead of actively following the total cohort. Data entry of questionnaires is only needed for cases and subcohort members, instead of the total cohort [8, 9]. Immediately after the NLCS-baseline measurement, the subcohort was sampled from the cohort, and actively followed up since 1986 for vital status and migration. The follow-up of the subcohort was 100% complete at 20.3 years of follow-up.

**Fig. 1:**
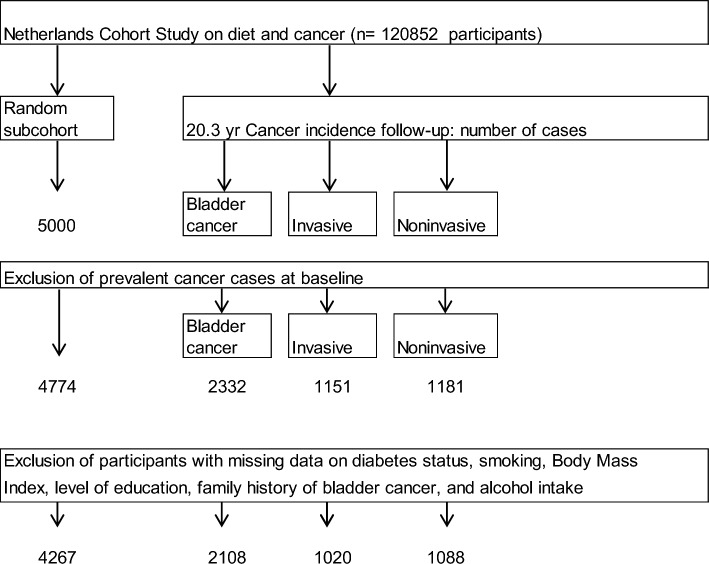
Flow diagram of the number of subcohort members and cancer cases on which analyses are based, Netherlands Cohort Study (NLCS)

Follow-up for cancer incidence in the entire cohort was established by annual record linkage with the Netherlands Cancer Registry and PALGA, the nationwide Dutch Pathology Registry [10]. Completeness of follow-up through record linkage with cancer registries and PALGA was estimated to be greater than 95% [11]. During 20.3 years of follow-up (September 17, 1986 until December 31, 2006), a total of 2332 bladder cancer cases (ICD-O-3 codes C67.0–C67.9) without prevalent cancer (except skin cancer) at baseline were detected. Bladder cancer cases were categorized in noninvasive (n = 1181, malignancy grade 2) and invasive (n = 1151, malignancy grade 3) subtypes. After excluding participants with prevalent cancer (except skin cancer) at baseline from the subcohort, 4774 subcohort members remained. Participants with missing values for the considered lifestyle factors and predefined confounders were excluded from the analysis. The current analysis included 1020 invasive and 1088 noninvasive bladder cancer cases, and 4267 subcohort members (Fig. 1).

### Exposure assessment

The 11-page baseline questionnaire measured dietary intake (including alcohol), detailed smoking habits, anthropometry, physical activity, chronic conditions and other risk factors related to cancer [8]. Regarding diabetes, the questionnaire contained the following questions: ‘Has a physician ever diagnosed you with diabetes mellitus and what was your age at that time?’ Participants could select the corresponding age category ranging from ‘younger than 30 years’, followed by 5-year age categories ranging from ‘30 to 34 years’ up to ‘65 to 69 years’. Based on previous epidemiological evidence, we determined that if participants indicated to have been diagnosed with DM after the age of 30 years, they were classified as having T2DM. Participants with probable Type 1 diabetes mellitus (age at diagnosis before 30 years) were considered as separate category. Duration of T2DM was calculated by subtracting the midpoint of the age category at diagnosis of T2DM from the age at baseline [12]. Participants were also asked to indicate ‘What medication they used longer than 6 months, for what condition(s) and in what period(s)?’. They could fill in the name of the medication and for what condition the medicine was used in their respective time period(s). The medication was classified according to the Anatomical Therapeutic Chemical (ATC) [13] from the World Health Organization Collaborative Centre for Drug Statistical Methodology. Antidiabetic medication was categorized as drugs based on “insulin and analogues” and “drugs lowering the blood glucose level (excluding insulin)” [14].

Tobacco smoking was addressed through questions on smoking status (never, ex, or current smoker) and inhalation for cigarette, cigar, and pipe smokers. Additional questions were asked on the ages at first and last exposure to smoking, smoking frequency, and duration for cigarette, cigar, and pipe smokers. Information on height (in cm) and weight at baseline (in kg) was also collected using the self-administered questionnaire, from which BMI (weight/height^2^) was calculated in kg/m^2^. The food-frequency questionnaire has been validated and tested for reproducibility [15, 16]. Habitual alcohol consumption during the year preceding baseline was assessed using questions on beer, red wine, white wine, sherry and other fortified wines, liqueur types containing on average 16% ethanol, and (Dutch) gin, brandy, and whiskey. Respondents who consumed alcoholic beverages less than once a month were considered non-users. Alcohol intake was calculated using the computerized Dutch food composition table [17].

### Statistical analysis

The distribution of the subcohort members and cases by diabetes exposure variables and various characteristics was examined by cross-tabulations and summary statistics. Hazard ratios (HRs) and 95% confidence intervals (95% CIs) for associations of diabetes exposure variables with incidence of bladder cancer were estimated using Cox proportional hazards models with follow-up duration as time variable. Participants (subcohort members and cases) with T1DM were excluded from analyses on T2DM, and vice versa. Person-years at risk for subcohort members were calculated from baseline until diagnosis of bladder cancer, death, emigration, loss to follow-up or end of follow-up, whichever came first. Standard errors were estimated using the Huber-White sandwich estimator to account for the increased variance because of subcohort sampling [18]. It was verified that the proportional hazards assumption was not violated using scaled Schoenfeld residuals [19] and -ln(-ln) survival plots.

For T2DM, the following exposure variables were assessed: T2DM status (no; yes), age at T2DM diagnosis (< 50 years; 50–< 55 years; 55+ years), duration of T2DM (< 5 years; 5–< 10 years; 10+ years), use of antidiabetic medication (no medication; use of blood glucose lowering drugs (excluding insulin); insulin and analogues treatment). For T1DM, only status could be assessed.

Analyses for overall, invasive and noninvasive bladder cancer were conducted for men and women separately, adjusted for age, as well as combined, adjusted for age and sex. In multivariable-adjusted survival analyses, the associations were adjusted for the following predefined (literature-based) confounders, which were included in the final multivariable-adjusted model independent of their effect on the estimated HRs: age at baseline (years), sex (men, women), smoking status (never, former, current), smoking frequency (number of cigarettes per day; centered), smoking duration (number of years; centered), BMI at baseline (< 18.5, 18.5–< 25, 25–< 30, ≥ 30 kg/m^2^), highest level of education (primary school or lower vocational (low), secondary school or medium vocational (medium), and higher vocational or university (high)), alcohol intake (g/day), family history of bladder cancer (no, yes). Tests for trends were assessed by fitting ordinal variables as continuous terms in the Cox regression models.

In addition to the main analyses, analyses of T2DM status and cancer risk were also stratified by age at baseline, smoking status, BMI, alcohol intake, level of education. (Numbers were too small for stratification on family history of bladder cancer). Interactions with these factors were tested using Wald tests and cross-product terms. Because diabetes could be a sign of subclinical cancer resulting in detection bias and reverse causality [20], sensitivity analyses were conducted, in which the main analyses were repeated after excluding cancers (and person-years) occurring in the first 2 years of follow-up,

Analyses were performed using Stata version 14; presented *P* values are two-sided, with *p* < 0.05 considered as statistically significant.

## Results

There were 1771 male incident bladder cancer cases (834 invasive, 937 noninvasive) and 337 female cases (186 invasive, 151 noninvasive) with complete data available for analyses, together with 2099 male and 2168 female subcohort members (Table 1). The number of subcohort members who reported a history of T2DM at baseline was 143 (prevalence 3.4%). The T2DM prevalence was somewhat higher among female subcohort members than in men: 3.5% versus 3.2%, respectively (Table 1). T2DM prevalence was higher among male (3.9%) and female (5.9%) invasive bladder cancer cases than among subcohort members, and lower among noninvasive bladder cancer cases (zero in female noninvasive cases). Male and female bladder cancer cases were on average slightly older at baseline, and reported higher alcohol intakes and higher smoking rates (also for frequency and duration) than subcohort members (Table 1). There were no clear differences between subcohort and cases according to BMI and education level in men and women. Cases reported somewhat more often a family history of bladder cancer. In participants with T2DM, the mean age at diagnosis of T2DM was somewhat higher in male subcohort members than in bladder cancer cases, but in women T2DM was diagnosed later in cases than in subcohort members. Particularly invasive bladder cancer cases more often reported use of antidiabetic drugs than subcohort members in both men and women (Table 1). For both sexes combined, the prevalence of T1DM was 0.2% (n = 7) among subcohort members, versus 0.4% (n = 4) in invasive and 0.2% (n = 2) in noninvasive bladder cancer cases.

**Table 1:** Baseline characteristics (means with SD, or percent) of bladder cancer cases and subcohort members with complete diabetes and covariable data, Netherlands Cohort Study

Characteristic	Men			Women		
	Subcohort	Bladder cancer cases		Subcohort	Bladder cancer cases	
		Invasive	Noninvasive		Invasive	Noninvasive
N	2099	834	937	2168	186	151
Age at baseline, mean (SD) (years)	61.3 (4.2)	62 (4.1)	61.7 (4.1)	61.4 (4.3)	61.9 (4.2)	61.6 (4.2)
Age at diagnosis cancer, mean (SD) (years)		73.4 (6.2)	73.2 (6.2)		74.5 (6.8)	73.7 (6.4)
BMI, mean (SD) (kg/m^2^)	25 (2.6)	25.0 (2.5)	25.1 (2.6)	25.1 (3.5)	25.0 (3.8)	25.0 (3.6)
Alcohol intake, mean (SD) (g/day)	14.9 (16.8)	17.9 (18.4)	16.9 (17.1)	5.7 (9.4)	7.4 (12.1)	6.4 (9.9)
Never smoker, n (%)	208 (9.9)	38 (4.6)	51 (5.4)	1290 (59.5)	90 (48.4)	73 (48.3)
Ever cigarette smokers only						
Smoking frequency, mean (SD) (cigarettes/day)	17.2 (10.6)	18.5 (10.7)	17.7 (10.4)	11.6 (8.4)	13.2 (8.6)	13.9 (9)
Smoking duration, mean (SD) (years)	33.7 (11.7)	37.4 (10.7)	35.8 (11.2)	27.9 (12.4)	31.8 (11.5)	32.1 (12.4)
University or higher vocational education, n (%)	396 (18.9)	155 (18.6)	183 (19.5)	194 (8.9)	13 (7)	10 (6.6)
Family history of bladder cancer, n (%)	15 (0.7)	7 (0.8)	8 (0.9)	31 (1.4)	3 (1.6)	5 (3.3)
History of diabetes						
Type 2 diabetes (T2DM), n (%)	67 (3.2)	32 (3.9)	28 (3)	76 (3.5)	11 (5.9)	0 (0)
Type 1 diabetes (T1DM), n (%)	2 (0.1)	3 (0.4)	2 (0.2)	5 (0.2)	1 (0.6)	0 (0)
Subjects with T2DM only						
Age at diagnosis of T2DM, mean (SD) (years)	54.4 (7.7)	53.6 (9.2)	52.9 (7.5)	54.8 (7.4)	60.2 (4.6)	N.A
Time since diagnosis T2DM, mean (SD) (years)	8.8 (7)	9.3 (8.8)	8.4 (6.3)	8.3 (6.3)	3.6 (2.7)	N.A
T2DM antidiabetic medication						
T2DM without antidiabetic medication, n (%)	34 (51.5)	15 (46.8)	18 (66.7)	39 (51.3)	1 (9.0)	N.A
T2DM with blood glucose lowering drugs,	28 (42.4)	12 (37.5)	7 (25.9)	28 (36.8)	10 (91.0)	N.A
excluding insulin, n (%)						
T2DM with insulin and analogues, n (%)	4 (6.0)	5 (15.6)	2 (7.4)	9 (11.8)	0 (0)	N.A.

Table 2 summarizes several baseline characteristics by T2DM status in male and female subcohort members separately (combined for T1DM because of low numbers). Compared to participants without DM, mean age at baseline and BMI were higher in both men and women with T2DM, and lower in those with T1DM, while mean alcohol intake was higher among men with T2DM, but lower in women with T2DM. (Men and women with T1DM had lower mean alcohol intake.) Compared to subjects without DM, men with T2DM were more often ever smokers and these ever smokers smoked on average more cigarettes for a longer period. This was not observed in women, where subjects with T2DM were more often never smokers. (Subjects with T1DM had a lower smoking frequency and duration, but these were mostly women.) Men and women with T2DM were less often highly educated, and more often reported a family history of bladder cancer compared to those without DM (Table 2).

**Table 2:** Baseline characteristics (means with SD, or percent) by diabetes mellitus status (Type 2 (T2DM) and Type 1 (T1DM)) in male and female subcohort members with complete diabetes and covariable data, Netherlands Cohort Study

Characteristic	Men (N)		Women (N)		Men and women (N)	
	No DM	T2DM	No DM	T2DM	No DM	T1DM
	2029	67	2087	76	4116	7
Women, n (%)					2087 (50.7)	5 (71.4)
Age at baseline, mean (SD) (years)	61.2 (4.2)	63.2 (4.0)	61.4 (4.3)	63.0 (4.2)	61.3 (4.2)	59.7 (4.0)
BM, mean (SD) (kg/m^2^)	25.0 (2.6)	25.5 (3.0)	25.0 (3.5)	26.2 (3.5)	25.0 (3.1)	23.5 (3.8)
Alcohol intake, mean (SD) (g/day)	14.8 (16.5)	18 (24.3)	5.8 (9.5)	3.8 (6.2)	10.3 (14.2)	3.6 (6.8)
Never smoker, n (%)	203 (10)	5 (7.5)	1233 (59.1)	54 (71.1)	1436 (34.9)	3 (42.9)
Ever cigarette smokers only						
Smoking frequency, mean (SD) (cigts/day)	17.1 (10.5)	19.8 (11.8)	11.6 (8.4)	12.2 (8.8)	15.3 (10.2)	9.3 (2.8)
Smoking duration, mean (SD) (years)	33.7 (11.7)	36.3 (10.4)	28 (12.4)	22.5 (11.6)	31.8 (12.2)	22.0 (13.2)
University or higher voc. education, n (%)	384 (18.9)	10 (14.9)	191 (9.2)	2 (2.6)	575 (14)	3 (42.9)
Family history of bladder cancer, n (%)	14 (0.7)	1 (1.5)	29 (1.4)	2 (2.6)	43 (1.0)	0 (0)

After excluding those with T1DM at baseline, 1016 invasive bladder cancer cases (831 men, 185 women) and 1086 noninvasive cases (935 men; 151 women), and 4260 subcohort members were available for T2DM analyses. Overall and subtype-specific associations between T2DM characteristics and bladder cancer risk are shown in Table 3 for men and women combined, and sex-specific associations in Table 4 (because there was significant interaction by sex for several exposures). While in age-sex-adjusted and in multivariable-adjusted analyses T2DM was not statistically significantly associated with overall bladder cancer in men and women combined (Table 3), there was a statistically significant association with risk of invasive bladder cancer with a multivariable-adjusted Hazard Ratio (HR) of 1.57 (95% CI 1.04–2.37) when comparing those with T2DM to those without DM; the test for interaction by sex was not statistically significant (*p* = 0.214). T2DM was not associated with noninvasive bladder cancer risk with a multivariable-adjusted HR of 0.99 (95% CI 0.63–1.56); there was significant interaction by sex because there were no female noninvasive cases with T2DM (*p* < 0.001). The cumulative incidence [21] of bladder cancer (calculated in accordance with the case-cohort design) is plotted against follow-up time in Supplementary Figure S1.

**Table 3:** Hazard Ratio of bladder cancer subtypes, according to diabetes characteristics in age-sex-adjusted and multivariable-adjusted analyses, Netherlands Cohort Study

Diabetes characteristics	Person- years subcohort	Overall bladder cancer			Invasive bladder cancer			Noninvasive bladder cancer		
		Cases	Age-sex-adjusted	Multivariable-adjusted	Cases	Age-sex-adjusted	Multivariable-adjusted	Cases	Age-sex-adjusted	Multivariable-adjusted
		n	HR (95% CI)	HR (95% CI)	n	HR (95% CI)	HR (95% CI)	n	HR (95% CI)	HR (95% CI)
T2DM status										
No DM	69,455	2031	1 (Ref)	1 (Ref)	973	1 (Ref)	1 (Ref)	1058	1 (Ref)	1 (Ref)
T2DM	2051	71	1.23 (0.87–1.73)	1.28 (0.90–1.82)	43	1.51 (1.02–2.24)	1.57 (1.04–2.37)	28	0.95 (0.61–1.50)	0.99 (0.63–1.56)
* P*-interaction by sex				0.944			0.214			< 0.001
Age at diagnosis of T2DM										
No DM	69,455	2031	1 (Ref)	1 (Ref)	973	1 (Ref)	1 (Ref)	1058	1 (Ref)	1 (Ref)
T2DM, diagnosed 55+ years	1136	41	1.34 (0.85–2.11)	1.45 (0.90–2.35)	29	1.90 (1.16–3.13)	2.11 (1.24–3.61)	12	0.78 (0.40–1.51)	0.83 (0.42–1.63)
T2DM, diagnosed 50–< 55 years	484	13	0.86 (0.42–1.77)	0.81 (0.38–1.71)	5	0.69 (0.25–1.87)	0.63 (0.23–1.76)	8	1.02 (0.44–2.36)	0.99 (0.42–2.32)
T2DM, diagnosed < 50 years	431	17	1.41 (0.68–2.91)	1.50 (0.72–3.12)	9	1.54 (0.66–3.59)	1.60 (0.67–3.81)	8	1.29 (0.53–3.15)	1.39 (0.57–3.41)
*P*-trend			0.323	0.269		0.147	0.140		0.847	0.740
* P*-interaction by sex				0.742			< 0.001			< 0.001
Time since T2DM diagnosis										
No DM	69,455	2031	1 (Ref)	1 (Ref)	973	1 (Ref)	1 (Ref)	1058	1 (Ref)	1 (Ref)
T2DM, < 5 years	676	29	1.59 (0.89–2.83)	1.6 (0.87–2.95)	21	2.35 (1.26–4.36)	2.39 (1.22–4.69)	8	0.86 (0.37–1.98)	0.85 (0.36–2.00)
T2DM, 5- < 10 years	657	20	1.18 (0.66–2.11)	1.25 (0.69–2.27)	9	1.07 (0.50–2.30)	1.17 (0.54–2.55)	11	1.28 (0.63–2.59)	1.33 (0.65–2.72)
T2DM, 10+ years	718	22	0.97 (0.54–1.76)	1.02 (0.56–1.88)	13	1.17 (0.59–2.32)	1.20 (0.59–2.44)	9	0.78 (0.36–1.71)	0.85 (0.38–1.87)
*P*-trend			0.553	0.417		0.217	0.179		0.783	0.953
* P*-interaction by sex				0.666			0.058			< 0.001
T2DM medication										
No DM	69,455	2031	1 (Ref)	1 (Ref)	973	1 (Ref)	1 (Ref)	1058	1 (Ref)	1 (Ref)
T2DM without antidiabetic drugs	1086	34	1.08 (0.67–1.74)	1.13 (0.70–1.84)	16	1.03 (0.57–1.88)	1.11 (0.60–2.04)	18	1.13 (0.63–2.01)	1.16 (0.65–2.08)
T2DM + blood glucose lowering drugs	757	29	1.33 (0.78–2.29)	1.35 (0.76–2.40)	22	2.08 (1.17–3.71)	2.06 (1.10–3.85)	7	0.63 (0.27–1.46)	0.65 (0.27–1.53)
T2DM + insulin and analogues	189	7	2.04 (0.71–5.84)	1.94 (0.61–6.17)	5	2.82 (0.90–8.80)	2.65 (0.76–9.18)	2	1.20 (0.25–5.91)	1.17 (0.22–6.19)
* P*-interaction by sex				< 0.001			< 0.001			< 0.001
T1DM										
No DM	69,455	2031	1 (Ref)		973	1 (Ref)		1058	1 (Ref)	
T1DM	106	6	3.14 (0.81–12.12)		4	4.42 (1.05–18.59)		2	1.99 (0.33–11.91)	
*P*-interaction by sex			0.726			0.949			< 0.001	

*DM* diabetes mellitus, *T2DM* type 2 diabetes mellitus, *T1DM* type 1 diabetes mellitus, *HR* hazard ratio

Multivariable analyses adjusted for: age at baseline (years), sex (men, women), smoking status (never, former, current), smoking frequency (number of cigarettes per day; centered), smoking duration (number of years; centered), BMI at baseline (< 18.5, 18.5–< 25, 25–< 30, ≥ 30 kg/m^2^), highest level of education (primary school or lower vocational (low), secondary school or medium vocational (medium), and higher vocational or university (high)), alcohol intake (g/day), family history of bladder cancer (no, yes)

**Table 4:** Hazard Ratio of bladder cancer subtypes, according to diabetes characteristics in age-adjusted and multivariable-adjusted analyses in men and women separately, Netherlands Cohort Study

Diabetes characteristics	Men							Women			
	Person- years subcohort	Invasive bladder cancer			Noninvasive bladder cancer			Person-	Invasive bladder cancer		
		Cases	Age-adjusted	Multivariable-adjusted	Cases	Age-adjusted	Multivariable-adjusted	years	Cases	Age-adjusted	Multivariable-adjusted
		n	HR (95% CI)	HR (95% CI)	n	HR (95% CI)	HR (95% CI)	subcohort	n	HR (95% CI)	HR (95% CI)
T2DM status											
No DM	32,160	799	1 (Ref)	1 (Ref)	907	1 (Ref)	1 (Ref)	37,295	174	1 (Ref)	1 (Ref)
T2DM	834	32	1.40 (0.88–2.24)	1.42 (0.88–2.30)	28	1.14 (0.71–1.85)	1.17 (0.72–1.89)	1216	11	1.94 (1.00–3.75)	2.19 (1.10–4.34)
Age at diagnosis of T2DM											
No DM	32,160	799	1 (Ref)	1 (Ref)	907	1 (Ref)	1 (Ref)	37,295	174	1 (Ref)	1 (Ref)
T2DM, diagnosed 55+ years	395	19	1.64 (0.88–3.06)	1.72 (0.89–3.33)	12	0.98 (0.49–1.99)	1.02 (0.50–2.07)	740	10	2.76 (1.35–5.65)	3.31 (1.54–7.12)
T2DM, diagnosed < 55 years	439	13	1.16 (0.58–2.32)	1.13 (0.56–2.31)	16	1.30 (0.68–2.48)	1.31 (0.68–2.51)	476	1	0.49 (0.07–3.65)	0.51 (0.07–3.87)
*P*-trend			0.265	0.284		0.484	0.452			0.208	0.134
Time since T2DM diagnosis											
No DM	32,160	799	1 (Ref)	1 (Ref)	907	1 (Ref)	1 (Ref)	37,295	174	1 (Ref)	1 (Ref)
T2DM, < 5 years	255	15	2.13 (1.00–4.55)	2.02 (0.91–4.50)	8	1.05 (0.43–2.56)	1.02 (0.42–2.51)	422	6	3.07 (1.23–7.69)	3.94 (1.54–10.10)
T2DM, 5+ years	580	17	1.08 (0.59–1.96)	1.12 (0.61–2.08)	20	1.18 (0.67–2.08)	1.24 (0.70–2.18)	795	5	1.34 (0.52–3.44)	1.43 (0.55–3.74)
* P*-trend			0.340	0.306		0.555	0.480			0.137	0.091
T2DM medication											
No DM	32,160	799	1 (Ref)	1 (Ref)	907	1 (Ref)	1 (Ref)	37,295	174	1 (Ref)	1 (Ref)
T2DM without antidiabetic drugs	441	15	1.18 (0.61–2.28)	1.25 (0.64–2.43)	18	1.34 (0.72–2.48)	1.37 (0.74–2.53)	645	1	0.34 (0.05–2.47)	0.38 (0.05–2.81)
T2DM + blood glucose lowering drugs	336	12	1.41 (0.67–2.96)	1.34 (0.61–2.94)	7	0.74 (0.31–1.79)	0.75 (0.31–1.82)	420	10	5.14 (2.39–11.09)	6.08 (2.75–13.43)
T2DM + insulin and analogues	38	5	5.08 (1.17–22.06)	4.18 (0.88–19.79)	2	1.96 (0.32–12.04)	1.74 (0.26–11.50)	151	0		

*DM* diabetes mellitus, *T2DM* type 2 diabetes mellitus, *HR* hazard ratio

Multivariable analyses adjusted for: age at baseline (years), smoking status (never, former, current), smoking frequency (number of cigarettes per day; centered), smoking duration (number of years; centered), BMI at baseline (< 18.5, 18.5–< 25, 25–< 30, ≥ 30 kg/m^2^), highest level of education (primary school or lower vocational (low), secondary school or medium vocational (medium), and higher vocational or university (high)), alcohol intake (g/day), family history of bladder cancer (no, yes)

The majority of the participants with T2DM had been diagnosed with DM at age 55 years or older, and less than 10 years before baseline. The duration of T2DM seemed significantly inversely related with risk of invasive bladder cancer, with the highest multivariable-adjusted HR of 2.39 (95% CI 1.22–4.69) observed in those diagnosed less than five years before baseline, compared to those without DM. For overall bladder cancer, a similar, but weaker, pattern was observed, while there was no association with noninvasive bladder cancer. An age at T2DM diagnosis of 55+ years was significantly associated with risk of invasive bladder cancer HR of 2.11 (95% CI 1.24–3.61) compared to those without DM, with no significant associations in the other age categories; however, there was significant interaction by sex (*P* < 0.001). Bladder cancer overall and noninvasive bladder cancer showed no significant associations with age at T2DM diagnosis (Table 3).

Regarding use of T2DM medication, those who used insulin or analogues showed the highest increased risk of invasive bladder cancer HR = 2.65 (95% CI 0.76–9.18), followed by those who used glucose-lowering drugs (excluding insulin) HR = 2.06 (95% CI 1.10–3.65). However, the HR in the insulin (or analogues) category was based on only 5 invasive cases, which may explain why this estimate is not statistically significant. There was no increased risk of invasive cancer for those with T2DM who did not use antidiabetic drugs. There was significant interaction of antidiabetic drug use by sex regarding invasive bladder cancer risk (*P* < 0.001). For overall bladder cancer, the same, but weaker pattern was seen; there was no association between antidiabetic drug use and noninvasive bladder cancer (Table 3). Analysis of T2DM duration by use of antidiabetic drugs among subcohort members revealed that the median T2DM duration in those who used glucose-lowering drugs was shorter than in those with T2DM without antidiabetic drugs (median 6 versus 7 years, respectively), but duration was longer among those who used insulin or analogues (median 8.5 years, data not shown). Thus, use of antidiabetic drugs was not particularly increased among those with T2DM duration < 5 years. Generally, age-sex-adjusted analyses and multivariable-adjusted analyses yielded comparable results for T2DM exposure characteristics.

Because there was significant interaction by sex for invasive bladder cancer, sex-specific analyses of T2DM characteristics and invasive bladder cancer are shown in Table 4. The sex-specific analyses indicated that the association between T2DM history and invasive bladder cancer was stronger (and significantly) positive in women than in men, with multivariable-adjusted HRs (95% CI) of 2.19 (1.10–4.34) in women versus 1.42 (0.88–2.30) in men. Analyses according to age at T2DM diagnosis and time since T2DM diagnosis showed significantly elevated risks of invasive bladder cancer in women with age at T2DM diagnosis of 55+ years HR = 3.31 (95% CI 1.54–7.12), and with duration of T2DM shorter than five years HR = 3.94 (95% CI 1.54–10.10). Because of lower case numbers, only two categories of age at diagnosis and duration were considered in sex-specific analyses. In men, the risks of invasive bladder cancer were also elevated in these same categories of age at diagnosis and duration of T2DM as in women, but not significantly (Table 4). Noninvasive bladder cancer risk was not associated with T2DM characteristics in men; in women there were no cases with noninvasive bladder cancer with T2DM. Regarding T2DM medication, women who used glucose-lowering drugs (excluding insulin) had a significantly increased risk of invasive bladder with a multivariable-adjusted HR of 6.08 (95% CI 2.75–13.43), compared to women without T2DM. In men, the highest risk of invasive bladder cancer was observed in those who used insulin (analogues) with a HR of 4.18, but this was not significant (based on only five cases). Men who used glucose-lowering drugs had no significantly increased risk (Table 4).

**Fig. 2:**
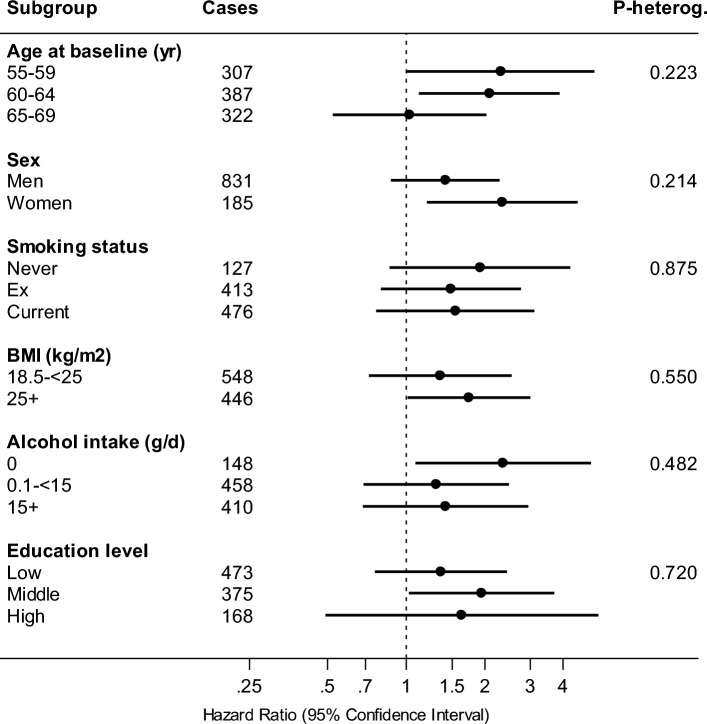
Hazard ratios and 95% CIs (error bars) of invasive bladder cancer, comparing participants with T2DM to nondiabetics, in subgroups of potential effect modifiers, Netherlands Cohort Study (NLCS). Multivariable analyses were adjusted for: age at baseline (years), sex (men, women), smoking status (never, former, current), smoking frequency (number of cigarettes per day; centered), smoking duration (number of years; centered), BMI at baseline (< 18.5, 18.5–< 25, 25–< 30, ≥ 30 kg/m^2^), highest level of education (primary school or lower vocational (low), secondary school or medium vocational (medium), and higher vocational or university (high)), alcohol intake (g/day), family history of bladder cancer (no, yes)

In sensitivity analyses, excluding cases occurring in the first two years of follow-up did not materially change the results (data not shown). To evaluate potential effect modification of the association between T2DM and invasive bladder cancer, subgroup analyses were conducted. Figure 2 shows associations between T2DM status and invasive bladder cancer risk, in subgroups of potential effect modifiers: age at baseline, smoking status, BMI, alcohol intake, level of education (family history of bladder cancer was too infrequent to investigate). Positive associations were seen in almost all subgroups, and there was no significant interaction.

After excluding those with T2DM at baseline, there were six incident bladder cancer cases (5 men, 1 woman; 4 invasive, 2 noninvasive) and 4124 subcohort members with complete data available for T1DM analyses. In age-sex-adjusted analyses (numbers were considered too small for multivariable-adjusted analyses), T1DM was not significantly associated with overall bladder cancer in men and women combined (Table 3), with a HR of 3.14 (95% CI 0.81–12.12) when comparing those with T1DM to those without DM; there was no significant interaction by sex. The association was stronger and statistically significant for invasive bladder cancer with a multivariable-adjusted Hazard Ratio (HR) of 4.42 (95% CI 1.05–18.59), with no significant interaction by sex. The HR of 1.99 for noninvasive bladder cancer (only two cases) was not significant.

## Discussion

In this large prospective study among Dutch men and women aged 55–69 years, the risk of invasive bladder cancer was 57% increased for participants with T2DM, compared to those without DM. Women showed a stronger positive association between T2DM and invasive bladder cancer than men, but interaction by sex was not statistically significant. Associations were stronger positive in those whose age at diagnosis of T2DM was 55 years or older, and in those diagnosed with T2DM less than five years before baseline. T2DM participants who used insulin or analogues showed the highest increased risk of invasive bladder cancer, followed by those who used glucose-lowering drugs (excluding insulin). No significant associations were observed between T2DM characteristics and noninvasive bladder cancer; however, no women with T2DM developed noninvasive bladder cancer. Although based on a small number of subjects with T1DM, invasive bladder cancer risk was strongly increased for participants with T1DM, compared to nondiabetics.

Previous meta-analyses of cohort and case–control studies on T2DM and bladder cancer risk [5, 6] found a modest positive association between T2DM status and bladder cancer with a summary RR of 1.35 [6]. While statistically significant positive associations were found in case–control and population cohort studies, there was no significant association in cohorts of diabetic patients [5]. The lack of association in the latter groups may partially attributable to underestimation of the true RR, because the general population, to which cohorts of diabetics are compared, also includes subjects with diabetes [5]. However, there was still severe heterogeneity in estimates, after stratification by study design [6]. In search of potential sources of heterogeneity, Zhu et al. [6] found that studies which adjusted for smoking showed considerably less and nonsignificant heterogeneity. Heterogeneity in estimates may also be due to studying overall bladder cancer instead of subtypes (invasive/noninvasive), and factors like sex differences, T2DM classification, and level of adjustment for confounders. Several subsequent cohort studies evaluated subtypes of bladder cancer. In the Iowa Women’s Health Study, an analysis of T2DM and bladder cancer risk suggested a stronger association in invasive than local (noninvasive) tumors [22]. In another large cohort study [23] an association was found between long-term T2DM, and insulin-use, and invasive bladder cancer risk. Other large cohort studies [24, 25], but not all [26] reported statistically significant positive associations between T2DM and bladder cancer in men and women. The UK biobank observed stronger associations in women, and also found positive associations with T2DM medication and longer duration, and with glycated hemoglobin levels [25]. Regarding cohort studies of diabetics, a Taiwanese cohort study [27] of diabetic patients found no association (without adjusting for important bladder cancer risk factors such as smoking), whereas a Korean study reported positive associations with T2DM after multivariable adjustment for confounders, and also with T2DM duration and impaired fasting glucose [28]. An Italian study [7] also found a positive association with bladder cancer in T2DM patients, but they only adjusted for age and sex. No distinction in bladder cancer subtypes was made in these latter studies.

Our results of a (modest) positive association with T2DM are in agreement with most earlier cohort studies, but our study indicates that this seems limited to invasive bladder cancer only, as was observed before [22, 23]. Like in the earlier meta-analysis [6], we found a stronger relationship in participants with T2DM duration less than five years, although some other recent studies reported stronger associations with longer duration [25, 28]. As we suggested earlier for colorectal cancer when we observed a higher risk with short duration of T2DM [12], the interpretation of results on the associations between the onset of T2DM and duration of exposure to T2DM on one hand and cancer risk on the other, is complex when considering a dynamic underlying pathway that involves varying degrees of insulin resistance and hyperinsulinemia, and associated alterations in IGF levels. First, intrinsic insulin levels vary from increased levels in the prediabetic stage to decreased levels in well-established T2DM as pancreatic β cells become more dysfunctional [29]. Second, from the time of T2DM diagnosis, various other factors such as medication [30, 31] and lifestyle changes (dietary changes, weight loss, increased physical activity) may influence the degree of insulin resistance and consequently serum levels of insulin. Taking into consideration this dynamic nature of the degree of insulin resistance, a history of T2DM may be viewed as a proxy for a temporary exposure to increased insulin and IGF levels and thus promotion of cell growth, which could increase the risk of cancer [12].

Regarding use of antihyperglycemic drugs, a recent review [32] concluded that using biguanides like metformin is associated with decreased total cancer risk, while sodium glucose cotransporter-2 inhibitors may increase bladder cancer risk [32]. However, SGLT2 inhibitors were not on the market at NLCS cohort baseline in 1986, as opposed to metformin, insulin and insulin analogues, and sulfonylureas (insulin secretagogues) which were available. The review also concluded that use of insulin (and analogues) is associated with a 50% increase in total cancer risk, compared to other antihyperglycemic drugs; mixed results were obtained for sulfonylureas [32]. Switching from only diet-treated to oral glucose-lowering drugs, and to insulin may indicate poorer glycemic control [7]. We found an increased risk of invasive bladder cancer in women who used glucose-lowering drugs (excluding insulin) and in men using insulin or analogues. This finding, and those of other studies on bladder cancer [23, 25] suggest that use of (certain) antidiabetic medications would increase invasive bladder cancer risk, independent of duration. Further studies are needed to investigate specific drugs in relation to invasive bladder cancer.

Several potential mechanisms underlying the increased bladder cancer risk in diabetes mellitus patients have been proposed. These include hyperinsulinemia (due to insulin resistance in T2DM or use of exogenous insulin) and increased levels of IGF-1 which stimulates cell proliferation and inhibits apoptosis [33, 34], and hyperglycemia affecting intracellular metabolism and impairing the immune system [35]. Also, glycated hemoglobin levels have been associated with bladder cancer risk [25]. Other potentially involved biological mechanisms include diabetes-related oxidative stress leading to altered cell energy metabolism and cell-cycle control, and possibly cancer development [36], and inflammatory cytokines which can influence cell apoptosis [37]. Diabetes mellitus is related to urinary tract infections [5, 38], which may be related to bladder cancer risk [39], but it might also be that more frequent urinary tract infections lead to earlier detection of bladder cancer. Since women have higher rates of these infections [40], this may (partially) explain the stronger associations between T2DM and bladder cancer in women compared to men. In the NLCS, the female-male difference in hazard ratios was observed for invasive bladder cancer. Remarkably, no female noninvasive bladder cancer cases occurred in participants with T2DM (or T1DM) during 20 years of follow-up. Apart from chance, the reasons why only invasive bladder cancers emerged in women with DM in this cohort are unclear; other cohort studies on this would be welcome. It has been shown that women are diagnosed with relatively more advanced bladder cancer than men [41, 42] (as was also seen in the NLCS), but the absence of noninvasive cases among female diabetics has not been reported before.

Regarding T1DM, the significantly positive association with (invasive) bladder cancer risk in the NLCS is in agreement with another recent cohort study from Italy [7]. Far less research has been done on T1DM and cancer risk than on T2DM. Whereas a recent meta-analysis reported significant associations between T1DM and various cancer types, no significant association with bladder cancer was seen [43]. However, the same review noted that most of the studies on T1DM and (bladder) cancer did not control for important risk factors that could act as confounders on the association between T1DM and cancer, for example smoking, obesity, education, alcohol [43]. Indeed, most of the included studies investigated cohorts of T1DM patients from diabetes registries with no information on these confounders. In the NLCS, exploratory multivariable-adjusted analyses controlling for smoking, obesity, alcohol and education showed stronger associations between T1DM and bladder cancer than age-sex-adjusted analysis but this was based on small numbers (data not shown). Because this observation is currently based on small numbers and thus might be due to chance (as in the recent Italian cohort study [7]), it should be studied in future large-scale cohorts with lifestyle and other confounder data whether T1DM is indeed associated with invasive bladder cancer, and whether multivariable adjustment for important risk factors such as smoking indeed yield stronger associations with T1DM. The largest study on T1DM and bladder cancer was conducted by Carstensen et al. [44], who compared cancer incidence in individuals with T1DM to the general population. They found no association with T1DM, but the study involved relatively young individuals (mean age at cancer diagnosis 51 years), there were no multivariable analyses controlling for known risk factors, and there was possible dilution bias due to using the general population as reference (containing T1DM and T2DM patients). In addition, they used an age at diagnosis of diabetes mellitus of 40 years as cutoff for T1DM instead of the usual age of 30 years.

Important strengths of the NLCS are the prospective design, a large sample size, availability of many detailed data on risk factors and lifestyle, and long follow-up, which also enabled subtype-specific analyses. Exclusion of cases occurring during the first two years of follow-up made detection bias and reverse causality less likely. The NLCS also has several limitations. While it has been suggested that the association between T2DM and bladder cancer might be due to unmeasured confounding [4], in the NLCS we have adjusted for many confounders (and found little difference with age-sex-adjustment only). We did not control for occupational risk factors for bladder cancer in this study, but an earlier bladder cancer analysis of the NLCS showed only weak associations with occupational risk factors such as exposure to paint components, polycyclic aromatic hydrocarbons, aromatic amines, and diesel exhaust in men [45]. It is also unlikely that occupational exposures act as confounders in the positive association between T2DM and bladder cancer in women. Nevertheless, residual confounding by unmeasured risk factors cannot be excluded. Also, the presence of survival bias cannot be excluded in this study. Survival bias can occur when a harmful exposure and age-related diseases are investigated in older populations, such as the NLCS in which subjects who were aged 55–69 years at baseline were recruited. This survival bias may lead to attenuated associations.

Because information on diabetes diagnosis and drug use was self-reported in the NLCS, this may have led to some misclassification. However, earlier studies have shown that self-reported (physician-diagnosed) diabetes is an accurate proxy for epidemiologic studies [46–48]. The validity of the questionnaire information on long-term drug use in the NLCS has been investigated before by comparing it to pharmacy records of dispensed drugs, indicating relatively good recall of long-term drug use [13]. Because there was no possibility in the NLCS to update information on diabetes status or antidiabetic medication use, or on confounders during follow-up, this may have resulted in attenuated associations. Some of the analyses on duration and antidiabetic drug use were based on small numbers, thus limiting the power of these analyses.

In conclusion, this cohort study showed that T2DM and possibly T1DM are positively associated with invasive bladder cancer risk, but not with noninvasive bladder cancer. Women showed a stronger positive association between T2DM and invasive bladder cancer than men.

Associations were stronger positive in those diagnosed with T2DM less than five years before baseline, and in T2DM participants using antidiabetic medication.

### Supplementary Information

Below is the link to the electronic supplementary material.

Supplementary file1 (PDF 236 KB)
