# Supplementary material for: Diabetes and the risk of bladder cancer subtypes in men and women: results from the Netherlands Cohort Study
Source: Eur J Epidemiol. 2024 Mar 16;39(4):379–91. doi: 10.1007/s10654-024-01100-0 (PMC11101497; doi:10.1007/s10654-024-01100-0)
Supplement: Supplementary file 1 — Supplementary file1 (PDF 236 KB) [file 10654_2024_1100_MOESM1_ESM.pdf]

SUPPLEMENTARY MATERIALS to:

European Journal of Epidemiology

Article:

P.A. van den Brandt "Diabetes and the risk of bladder cancer subtypes in men and women; results from the Netherlands Cohort Study"

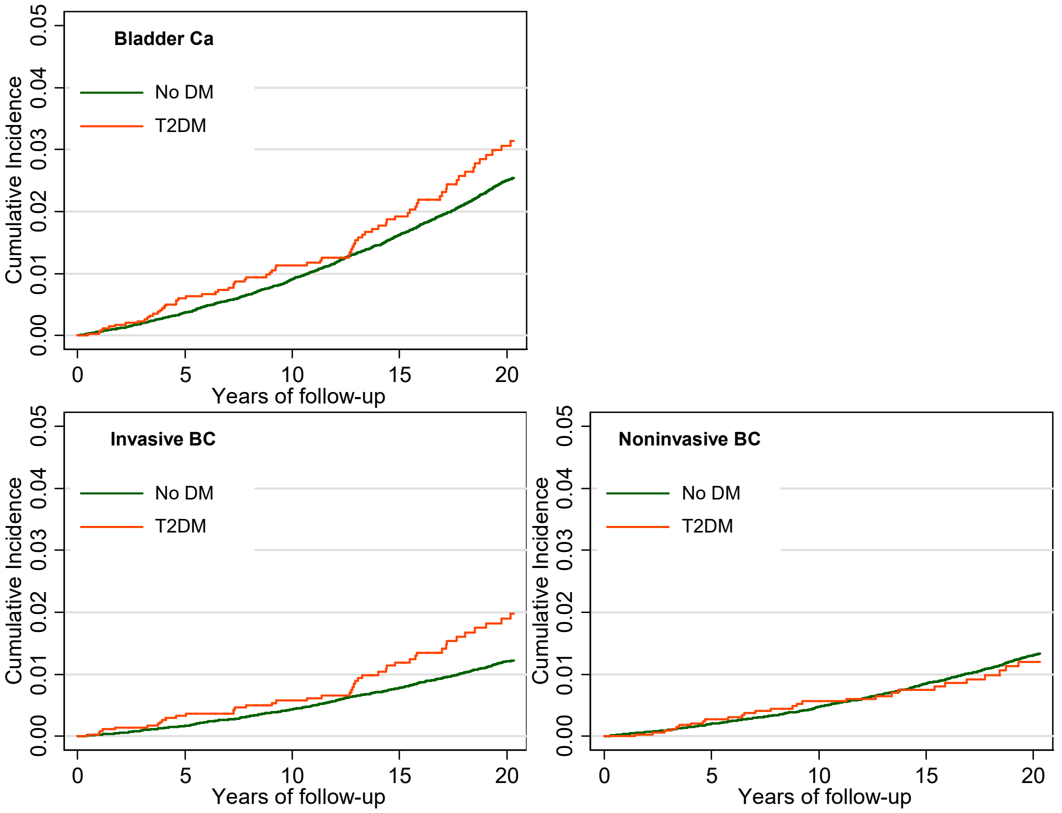

Supplementary Figure S1. Cumulative incidence (proportion) of overall bladder cancer, invasive and noninvasive bladder cancer, according to Type 2 diabetes mellitus (T2DM) status, Netherlands Cohort Study.
